# Dental Exostosis

**Published:** 1852-10

**Authors:** 


*Dental Exostosis.*—The most remarkable specimen of dental exostosis which we have ever seen was recently sent to us for the Museum of the Baltimore College of Dental Surgery, by Dr. V. M. Swayze of Easton, Penn., a graduate of the Institution. It is about the size of a hickory nut, and as every part of the tooth, except the apex of the root, is involved, the formation of it must have been simultaneous with the ossification of the pulp. It must also have been developed very rapidly, as it broke up and forced itself through the enamel membrane in every direction, and yet, it would seem, that the functions of this organ were performed, as nodules of enamel are interspersed through the greater portion of the exostosis. The tooth is a lower dens sapientiæ, and was extracted a few months since by the gentleman who sent it to us.

